# Investigating the Associated Factors of Mood Disorders and Fatigue in Multiple Sclerosis Patients With Cognitive Impairment: A Cross‐Sectional Study

**DOI:** 10.1002/hsr2.71457

**Published:** 2025-11-06

**Authors:** Behnaz Sedighi, Parya Jangipour Afshar, Simin Jafari

**Affiliations:** ^1^ Neurology Research Center Kerman University of Medical Sciences Kerman Iran; ^2^ HIV/STI Surveillance Research Center, and WHO Collaborating Center for HIV Surveillance, Institute for Futures Studies in Health Kerman University of Medical Sciences Kerman Iran

**Keywords:** cognitive impairment, fatigue, Iran, mood disorders, multiple sclerosis

## Abstract

**Background and Aims:**

This study aimed to investigate the factors associated with mood disorders and fatigue in multiple sclerosis patients with cognitive impairment.

**Methods:**

This cross‐sectional study included 70 out of 155 MS patients exhibiting cognitive impairment (CI) from a referral clinic in Kerman, Iran, between 2023 and 2024. The Persian‐adapted Brief International Cognitive Assessment for MS (BICAMS) tests, which include the California Verbal Learning Test (CVLT‐II) to assess auditory or verbal episodic memory, the Symbol Digit Modalities Test (SDMT) to assess visual processing speed and working memory have been used. Independent *t*‐test compared CI between patients with and without CI, and linear regression analyzed factors associated with mood disorder and fatigue.

**Results:**

Out of 70 patients, 61 (87.1%) were women. The most frequently reported symptoms were fatigue and mood disorders. The mean of BICAMS domains in patients with CI was significantly lower than in non‐CI patients (*p* < 0.05). And the Symbol Digit Modality Test (SDMT) was the most frequently impaired domain (63.3%). A regression analysis showed a positive association of age (B = 0.346, *p* < 0.001), MS duration (B = 0.245, *p* < 0.001), and Expanded Disability Status Scale (EDSS) (B = 0.637, *p* < 0.001) with mood disorders. Furthermore, woman participants exhibited greater fatigue intensity than men (B = 0.260, *p* = 0.005). Additionally, as EDSS increased, fatigue intensity increased (B = 0.259, *p* < 0.001).

**Conclusion:**

Information processing speed and working memory were the most affected cognitive domains in MS patients. Furthermore, mood disorders were more prevalent in older patients and those with higher EDSS, while fatigue was more pronounced in women and those with higher EDSS. Therefore, it is of higher importance to address the issue of CI and its association with fatigue and depression, particularly in the Iranian people.

## 1 Introduction

Cognitive impairment (CI) refers to a common set of signs and symptoms that have a significant impact on quality of life (QOL), work performance, and social functioning. These symptoms typically involve multiple mental functions, such as complex attention, memory, information processing speed, or executive function. Schreiner et al. [1, 2].

Multiple sclerosis (MS) is a chronic inflammatory disease of the central nervous system (CNS) that primarily affects young adults. It damages the brain and spinal cord and affects approximately 2.8 million people worldwide, manifesting a variety of specific and nonspecific symptoms [3, 4, 5, 6]. One of the most impactful nonspecific symptoms is CI, which significantly affects the daily lives of MS patients and can occur at various stages of the disease [1].

CI is a persistent and complex feature of CNS disorders and autoimmune diseases. It is often overlooked in the clinical evaluation of patients with MS [7]. The presentation of CI in MS patients varies throughout their lifetime and can be challenging to distinguish from other age‐related cognitive changes in older individuals. It is a common symptom in MS, with a prevalence of 50%–60%, significantly impacting patients' QOL, work performance, and social functioning [2, 7, 8]. Although CI is more frequently observed in the progressive forms of MS, it can also occur in the early stages of the disease, including clinically isolated syndrome (CIS). Lakin et al. [1, 2].

Alongside CI, two other often invisible symptoms fatigue and mood disorders are highly prevalent in MS and further complicate disease management. Fatigue, reported in up to 80% of patients according to the National MS Society, is a multifaceted symptom with cognitive, emotional, and physical components that can significantly interfere with daily activities, work performance, and overall QOL. Lakin et al. [1]. Similarly, mood disorders, particularly depression and anxiety, occur at disproportionately high rates in MS compared to the general population. Depression affects approximately 30.5% of people with MS, compared to 7% in the general population, while anxiety is reported in 22.1% of MS patients, compared to 19% in the general population. These elevated rates reflect both neurobiological changes associated with MS and the psychosocial burden of living with a chronic illness [1, 9].

Although fatigue [10, 11, 12] and mood disorders [10, 11] are known to contribute to cognitive performance deterioration, the relationships between these factors and cognitive efficiency remain poorly understood. Particularly, there is a notable lack of research focusing on these factors, especially within the Iranian context. The current study aimed to investigate the links among CI, mood disorders, and fatigue, as well as to evaluate the factors influencing fatigue and mood disorders in MS patients with CI in Kerman Province, Iran. The result can improve treatment plans, identify key factors influencing CI, enhance patients' QOL, and support better work and social adaptation.

## 2 Materials and Methods

### 2.1 Study Design and Participants

This cross‐sectional study included 70 out of 155 MS patients who exhibited CI from a referral clinic in Kerman, Iran, between 2023 and 2024. The patients included in the study did not have any other neurological disease, did not have CIS, were able to read and write, and were fluent in Persian. Patients with a learning disability, chronic comorbidities (e.g., diabetes, congestive heart failure, psychiatric or mental disorders, genitourinary pathologies), receiving corticosteroid pulse therapy or MS relapse within 12 weeks of the assessment, presence of physical impairment that could affect neuropsychological (NP) tests, and those who did not consent to participate were excluded from the study. The final diagnosis of MS in this registry was confirmed by neurologists who are affiliated with the Shafa Hospital and the Kerman University of Medical Sciences, based on the revised McDonald criteria (2017) [13]. This study was approved by the Ethics Committee of Kerman University of Medical Sciences (IR. KMU. REC.1402.271). Written informed consent was obtained from all the participants.

### 2.2 Neuropsychological Assessment

The Persian‐adapted Brief International Cognitive Assessment for MS (BICAMS) tests, which are used for neuropsychological assessment, are a valid instrument for MS patients. The tests include the California Verbal Learning Test (CVLT‐II) to assess auditory or verbal episodic memory, the Symbol Digit Modalities Test (SDMT) to assess visual processing speed and working memory, and the Brief Visuospatial Memory Test‐Revised (BVMT‐R) to assess visual or spatial episodic memory. Impairment is defined as a score that is at least 1.5 standard deviations below the mean normative value for each cognitive test. The reliability of the Persian version of the questionnaire was assessed and confirmed by Eshaghi et al. [14], who found that it exhibited good discriminant validity and reliability.

### 2.3 Outcomes Measurement

The demographic and clinical information form is utilized to assess the age, gender, education level, employment status, place of residence (urban or rural), the absence of common comorbidities (complications accompanying MS other than diabetes, hypertension, cardiovascular, cerebrovascular, and respiratory diseases), tobacco, alcohol, and substance abuse (opioids, heroin, methamphetamine, or other stimulants), and the patient's clinical conditions, including the duration of the disease, EDSS, fatigue, psychiatric symptoms, pain, sexual disorder and sphincter disorder.

The fatigue severity scale (FSS) was employed to assess fatigue. The FSS is a unidimensional questionnaire comprising nine statements that evaluate the severity and impact of fatigue in MS patients with cognitive impairment. The items are scored on a scale of 1 to 7, with 1 representing “completely disagree” and 7 representing “completely agree” and the cut‐off score for fatigue was set to be ≥ 4. The Persian version of the questionnaire has been reported to have a reliability and validity of 93% and 96%, respectively, by Shahvarughi‐Farahani et al. [15].

The mood disorder utilized the Patient Health Questionnaire‐9 (PHQ‐9), which assigns a score of “0” (not at all) to “3” (nearly every day) to each of the nine Diagnostic and Statistical Manual of Mental Disorders, Fourth Edition (DSM‐IV) criteria. Scores of 5, 10, 15, and 20 represent cut‐points for mild, moderate, moderately severe, and severe mood disorder, respectively (0–4: None‐minimal, 5–9: Mild,10‐14: Moderate,15–19: Moderately severe, 20–27: Severe.). The Persian version of the questionnaire by Ardestani et al. has been found to exhibit high levels of validity and reliability, with Cronbach's alpha values ranging from 91% to 93% [16].

The degree of disability experienced by patients with MS was evaluated using the EDSS. The score can range from 0, indicating a normal neurological state, to 10, which represents MS‐induced death. The EDSS score ranges from 0 to 10, with 0–2.5 indicating mild disability, 3–5 indicating moderate disability, and 5.5–10 indicating severe disability. Kurtzke [17].

All questionnaires were completed by the patients in a calm and private place under the supervision of a neurologist, and the EDSS was evaluated by a neurologist. The data were stored and analyzed anonymously.

### 2.4 Statistical Analysis

To describe the data, descriptive statistics such as number and percentage were used for qualitative variables, and mean and standard deviation for quantitative variables. An independent t‐test was applied to compare CI dimensions between patients with and without CI. To identify factors associated with mood disorder and fatigue, we performed linear regression analysis using the backward elimination method, which was selected to obtain a parsimonious model by systematically removing nonsignificant predictors. Before running the analyses, assumptions of normality, multicollinearity (using VIF), residual homoscedasticity, and model fit diagnostics were examined and confirmed. A significance level of 0.05 was applied, and analyses were conducted using SPSS version 26.

## 3 Result

### 3.1 Descriptive

According to the data, 61 (87.1%) of the 70 patients with CI were women. Most of these individuals 46 (65.7%) were over 30 years old and unemployed 46 (65.7%). A small percentage had physical disease (22.9%) or addictions (7.1%). The majority of patients had a mild EDSS 60 (85.7%), and the most common symptoms they reported were fatigue and mood disorders. (Table 1).

**Table 1:** Demographic characteristics of patients with cognitive disorder (*N* = 70).

Variables		Number	Percent
**Age**	< 30	21	30.0
	≥ 30	46	65.7
**Gender**	Man	9	12.9
	Woman	61	87.1
**Education (years)**	< 12	12	17.1
	12–16	39	55.8
	> 16	19	27.1
**Job**	Unemployed	46	65.7
	Self‐employed	14	20.0
	Employed	7	10.0
**Location**	Urban	66	94.2
	Rural	1	1.4
**Physical disease**	No	54	77.1
	Yes	16	22.9
**Addiction**	No	65	92.9
	Yes	5	7.1
**EDSS**	Mild (0–2.5)	60	85.7
	Moderate (3–5)	9	12.9
	Sever (5.5–10)	1	1.4
**Type of drug**	Injectable	6	8.6
	Anti CD20	30	42.8
	S1P receptor modulator (Fingolimod)	3	4.3
	Natalizumab	0	0
	Oral (Dimethyl fumarate, Teriflunomide)	20	28.6
	None	11	15.7
**Type of problem** a	Fatigue	62	88.6
	Mood disorders	49	70.0
	Pain	17	24.3
	Sexual disorder	16	22.9
	Sphincteric	11	15.7

*Note:* Missing cases may result in a low number and percentages.

^a^ The number of patients who have each of these symptoms has been reported. Each individual may experience one or more symptoms.

The individuals' average MS disease duration was 9.5 ± 7.2 years, ranging from 6 months to 24 years. (Table 2).

**Table 2:** Mean and standard deviation of variables related to patients with cognitive impairment.

	Cognitive impairment (*N* = 70)		
Variables	Mean ± SD	Min	Max
Age (year)	38.3 ± 10.5	25	60
The duration of MS (years)	9.5 ± 7.2	0.5	24
EDSS	1.2 ± 1.5	0	7
Fatigue	4.62 ± 0.7	3.33	5.78
Mood disorder	10.06 ± 5.0	4	20

The mean of BICAMS domains in patients with CI was significantly lower than in non‐CI patients (*p* < 0.05) (Table 3).

**Table 3:** Mean and standard deviation of each task of BICAMS and the cutoff of the normative data.

		Cognitive impairment				Noncognitive impairment			*p* value
Cognitive test	Cutoff [14]	Mean ± SD	Percent	Min	Max	Mean ± SD	Min	Max	
CVLTII‐TOTAL	42.62	38.93 ± 5.6	46.6	23	42	51.50 ± 5.6	43	65	0.007
SDMT	30.86	23.74 ± 3.9	63.3	18	29	45.14 ± 8.7	31	63	< 0.001
BVMT‐R‐ TOTAL	13.94	7.75 ± 0.5	8.4	7	8	26.14 ± 4.9	19	36	0.001

### 3.2 Analytical

A regression analysis of MS patients with CI found a positive association of age (B = 0.346, 95% CI: 0.124–0.569; *p* < 0.001), MS duration (B = 0.245, 95% CI: 0.091–0.537; *p* < 0.001), and EDSS (B = 0.637, 95% CI: 0.493–0.780; *p* < 0.001) with mood disorder. These findings indicate that higher values of these factors are associated with greater levels of mood disorder. Addicted patients showed higher mood disorder scores than non‐addicts (B = 0.444, 95% CI: 0.142–0.747; *p* < 0.001), and patients with a physical disease also reported higher mood disorder scores compared to those without a physical disease (B = 0.467, 95% CI: 0.258–0.675; *p* < 0.001). Moreover, women reported greater fatigue intensity compared to men (B = 0.260, 95% CI: 0.107–0.412; *p* = 0.001), and higher EDSS scores were positively associated with higher fatigue intensity (B = 0.259, 95% CI: 0.013–0.516; *p* < 0.001). (Table 4).

**Table 4:** Factors influencing Quality of life and Fatigue intensity in patients with cognitive impairment with linear regression.

Variables	Unstandardized coefficient (B)	95% Confidence Interval	*p* value
**Mood disorder**			
Age	0.346	0.124, 0.569	< 0.001
The duration of MS (years)	0.245	0.091, 0.537	< 0.001
Physical disease	0.467	0.258, 0.675	< 0.001
Addiction	0.444	0.142, 0.747	< 0.001
EDSS	0.637	0.493, 0.780	< 0.001
**Fatigue intensity**			
Gender (women)	0.260	0.107, 0.412	0.005
EDSS	0.259	0.013, 0.516	< 0.001

## 4 Discussion

The results of this study indicated that 70 out of 155 (45.2%) patients with MS had CI. The majority of patients with CI were women and had mild EDSS. The most common symptoms that they complained of were fatigue and mood disorders. The mean of BICAMS domains in patients with CI was significantly lower than in non‐CI patients.

MS patients with CI demonstrated that age, MS duration, and EDSS had a significant positive association with mood disorder. Additionally, women and patients with higher EDSS scores were more likely to experience fatigue.

In this study, the percentage of MS patients with CI was 45.2% which is higher than the Eteghad et al. study which reported the rate of CI was 30.4% in Tabriz, Iran [18]. However, the result was approximately in line with some studies that found CI in MS patients about 43%–44% [19, 20, 21, 22]. Also, a study using Minimal Assessment of Cognitive Function in Multiple Sclerosis (MACFIMS) found that 65.4% of MS patients had CI [23], which is higher than our ratio. The variability of CI in MS patients across the literature has been reported to range from 30% to 70%. This discrepancy could be attributed to methodological differences between studies, study design, data source, and demographic and clinical information of MS patients, including age, education level, cultural differences, disease duration, and EDSS score. In this study, the SDMT subtype, which represents visual processing speed and working memory, was the most frequently impaired domain (63.3%), in line with several previous studies that concluded the SDMT was the best test for detecting CI in MS patients, representing the most affected cognitive domains as information processing speed, verbal/visual memory and executive function [18, 24, 25].

In the current study, the majority of MS patients with CI exhibited mild EDSS. There was a positive and significant association between EDSS and disease duration, indicating that longer disease duration is linked to greater disability. These aligns with Benedict et al. showing that patients with mild EDSS and long disease duration can present with CI [26].

Our sample was predominantly female and had mostly low Expanded Disability Status Scale (EDSS) scores, indicating a relatively functionally intact group. While the high proportion of women aligns with the known epidemiology of MS, the predominance of low disability levels may limit the generalizability of our findings to patients with more severe clinical phenotypes. Future studies including a broader range of disability levels and a more balanced gender distribution are warranted to confirm whether these associations hold across diverse MS populations.

The findings of the current study revealed a significant positive association between age, disease duration, and EDSS scores with mood disorders in MS patients experiencing CI. These findings indicate that higher age, longer disease duration, and greater disability are associated with more severe mood disorders and poorer QOL in MS patients. This aligns with the findings of Mohammadi et al. [27], which highlighted similar findings. The observed relationship may be attributed to the cumulative effects of brain damage over time. As patients age and the disease progresses, the impact of MS‐related plaques on the brain becomes more pronounced. This progressive neural damage can disrupt emotional regulation and cognitive functions, contributing to the development of mood disorders.

Additionally, several other factors may exacerbate mood disorders and the decline in QOL in MS patients. These include physical disability, as reflected in higher EDSS scores, the side effects of treatment medications, and various socio‐cultural and economic conditions. Geographic location and residency‐related stressors, along with the severity and progression of MS itself, further compound the risk of mood disturbances. Collectively, these factors underscore the multifaceted challenges faced by MS patients and the importance of a holistic approach to their care [28, 29, 30].

The association between cognitive impairment, fatigue, and mood disorders in MS may stem from shared disruptions in fronto‐subcortical and limbic networks, which support both executive and emotional functions. Damage to these circuits—frequently affected in MS—could explain the overlap of symptoms, as supported by recent neurobiological and developmental research [31, 32, 33].

Beyond these circuits, emerging evidence highlights the role of large‐scale brain network disconnection, particularly in the default mode, salience, and executive control networks, which are increasingly recognized as central to MS‐related cognitive and emotional dysfunction. Disruption in the default mode network has been linked to impaired self‐referential processing and fatigue, while alterations in the salience and executive control networks may underlie attentional lapses, reduced cognitive flexibility, and vulnerability to mood disturbances. Integrating these insights suggests that the overlap of CI, fatigue, and mood disorders may reflect a broader neurofunctional model of MS‐related network disconnection. Future studies incorporating neuroimaging biomarkers could help validate this framework and clarify the pathways through which neural disconnection drives symptom clustering in MS [34].

Despite the absence of extensive literature on the factors influencing mood disorders and fatigue in MS patients with CI, this study identified a significant positive association between EDSS and mood disorders. In contrast, some studies did not establish a clear relationship between EDSS and mood disorders, suggesting variability in findings across different populations and methodologies. Provinciali et al. [35, 36]. Nevertheless, numerous studies have demonstrated that physical disabilities and limitations in activities of daily living are strongly linked to psychological consequences, such as low self‐esteem and diminished self‐confidence. These psychosocial challenges, in turn, contribute to the development of mood disorders and negatively impact QOL. Arnett et al. [28, 36]. The psychological burden of reduced autonomy, combined with the social isolation and dependency often experienced by patients with greater physical disability, further underscores the complex interplay between physical and psychological symptoms in MS. These findings highlight the importance of integrated care strategies that address both physical and mental health needs to improve overall patient outcomes.

The findings of this study also indicated that gender and EDSS scores were significantly associated with fatigue in MS patients with CI. Specifically, women and patients with higher EDSS scores were more likely to experience fatigue. These results are consistent with the findings of Mohammadi et al. and Alsaeed et al. Mohammadi et al. [27, 37], as well as studies conducted in Western Europe and North America [38], which reported that the majority of MS patients with fatigue were women, from a biological perspective, hormonal differences, particularly those related to estrogen and progesterone, might play a role in the severity of fatigue symptoms in women, for instance, has neuroprotective properties and modulates immune responses, which may be disrupted in MS [39]. Additionally, psychological factors such as greater levels of stress, depression, and anxiety may affect women more profoundly, leading to heightened fatigue intensity. Social factors, such as caregiving roles or societal expectations, may also contribute to the experience of fatigue, particularly in women. It should be noted, however, that the study sample was predominantly female and mostly comprised patients with low EDSS scores, which may limit the generalizability of these findings to patients with more severe MS phenotypes.

Additionally, Fidao et al. Fidao et al. [40] observed a higher prevalence of fatigue among MS patients with increased EDSS scores. However, the findings of this study were not entirely consistent with those of other research, which reported differing associations between fatigue and these variables [41, 42]. These discrepancies may be attributed to several factors, including the side effects of treatment medications, differences in socio‐cultural and economic conditions, geographic location, and residency‐related stressors. Variations in study populations, methodologies, and definitions of fatigue may also contribute to these contrasting findings.

Overall, these results highlight the multifactorial nature of fatigue in MS patients and underscore the need for tailored management approaches that consider individual demographic, clinical, and socio‐environmental factors.

To further clarify these complex associations and explore potential causal pathways, future longitudinal or prospective cohort studies are recommended.

## 5 Conclusion

In conclusion, the ratio of CI was found to be significantly higher in MS patients, particularly in the studied population of Kerman province, Iran. The most affected domain was SDMT, which evaluates processing information speed. Furthermore, mood disorders were more prevalent in older patients and those with higher EDSS, while fatigue was more pronounced in women and those with higher EDSS. These findings emphasize the importance of addressing cognitive impairment and its association with fatigue and mood disorders, particularly within this regional population. Although cognitive impairment is often overlooked in MS patients referred to clinics, it is crucial to recognize and manage this issue to improve overall quality of life.

## 6 Limitation

The present study was subject to certain limitations. First, the sample size was relatively small, predominantly female, and mostly comprised patients with low EDSS scores, which may limit the generalizability of the findings to patients with more severe MS phenotypes. Second, BICAMS was used because MACFIMS was time‐consuming and would have restricted the sample size. Third, the fatigue and mood disorder questionnaires were completed in MS patients with cognitive impairment, and no previous study has investigated the association between demographic and clinical characteristics and mood disorders and fatigue in this population. Fourth, this study was cross‐sectional, which limits the ability to draw causal inferences. Finally, some potential confounding factors, such as clinical, and socio‐environmental variables, were not fully controlled, which could have influenced the observed associations. Future longitudinal or prospective cohort studies are warranted to better elucidate the temporal and causal relationships among these factors.

## Author Contributions

**Behnaz Sedighi:** Conceptualization; Supervision; Writing – review and editing; Methodology. **Parya Jangipour Afshar:** Software; Visualization; Writing – original draft; Formal analysis. **Simin Jafari:** Project administration; Data curation; Writing – original draft; Visualization.

## Ethics Statement

This study was approved by the Ethics Committee of Kerman University of Medical Sciences (IR. KMU. REC.1402.271). Written informed consent was obtained from all the participants.

## Conflicts of Interest

The authors declare no conflicts of interest.

## Data Availability

The data that support the findings of this study are available from the corresponding author upon reasonable request.
